# Tool Wear Mechanism and Grinding Performance for Different Cooling-Lubrication Modes in Grinding of Nickel-Based Superalloys

**DOI:** 10.3390/ma16093545

**Published:** 2023-05-05

**Authors:** Chunyou Liang, Yadong Gong, Linhu Zhou, Yang Qi, Huan Zhang, Jibin Zhao

**Affiliations:** 1 School of Mechanical Engineering and Automation, Northeastern University, Shenyang 110819, China; 2 ChangChun Railway Vehicles, ChangChun 130062, China; 3 Technology Equipment and Intelligent Robot Laboratory, Shenyang Institute of Automation, Chinese Academy of Sciences, Shenyang 110169, China

**Keywords:** grinding, tool wear, cooling-lubrication, nickel-based superalloys, grinding performance

## Abstract

Tool wear introduced during grinding nickel-based superalloys was identified as a significant factor affecting the production quality of aero-engine industries concerning high service performance and high precision. Moreover, uncertainties derived from the various cooling-lubrication modes used in grinding operations complicated the assessment of grinding preformation. Therefore, this work investigated the tool wear mechanisms in grinding nickel-based superalloys that adopted five cooling-lubrication modes and investigated how the wear behaviors affected grinding performance. Results showed that chip-deposits covered some areas on the tool surface under dry grinding and accelerated the tool failure, which produced the highest values of tangential force, 7.46 N, and normal force, 14.1 N. Wedge-shape fractures induced by indentation fatigue were found to be the predominant wear mechanism when grinding nickel-based superalloys under flood cooling mode. The application of minimum quantity lubrication-palm oil (MQL-PO), MQL-multilayer graphene (MQL-MG), and MQL-Al_2_O_3_ nanoparticles (MQL-Al_2_O_3_) formed lubricity oil-film on the tool surface, which improved the capacity of lubrication in the tool–workpiece contact zone and provided 37%, 30%, and 52% higher coefficient of friction than dry mode, respectively. The results of this study demonstrate that lubricated oil-film produced by MQL modes reduces the possibility of fractures of cubic boron nitride (CBN) grits to some extent.

## 1. Introduction

Nickel-based superalloys are considered a key material system for improving gas turbines of aero-engines’ performance due to that they retain their strength and chemical properties at high working temperatures [1]. These materials have a complex of properties, such as low thermal conductivity, which leads to high heat generation in the abrasive grits–workpiece contact zone [2]; intense thermal affinity to the abrasive tool materials inducing the adhesion of alloy materials to tool surfaces [3]; work hardening; and existence of hard abrasive particles involving carbides, silicates, and oxides, causing significant tool wear. Therefore, Aspinwall et al. [4] considered that grinding of nickel-based superalloys is one of the most challenging machine areas.

Grinding is an abrasive manufacturing process for achieving high precision, close assembly tolerances, and high surface quality. However, grinding tool wear generated by the random fractures or dulling in abrasive grits during the grinding process, as described by Herman and Krzos [5], changes the surface quality of components. In particular, the grinding employing improper cooling-lubrication modes induces the hastening of tool wear involving wheel loading, thermal cracks, and clogging on abrasive grits, which results in the deterioration of surface finishing, as reported by Liang et al. [6]. Kuntoglu [7] reported that tool wear spoiling the main tool geometry can reduce the ability to form chips and increase the friction between the machined tool and workpiece. Hwang et al. [8] found that the rapid tool wear could not produce consistent parts finishing, continuous grit dulling, and progressive increase of grinding forces and specific energy that resulted in the rapid grinding tool failure. Therefore, conditions of tool wear can play a crucial role in determining a material’s ability to withstand service loading environments; its understanding is particularly relevant from an industrial perspective within the manufacture of high-quality components in the aerospace field.

Wear of grinding tools in grinding of nickel-based superalloys has been reported on in the available literature. Sunarto and Ichida [9] reported the properties of tool wear in creep feed grinding of nickel-based superalloys employing conventional flood cooling, describing that new cutting edges were provided by microfractures taking place on the tip of abrasive grits. The experimental results presented an improvement in the performance of grinding tools arising from such fracture characteristics of abrasive grits. Zhou et al. [10] characterized tool wear by microgrinding nickel-based superalloys employing the dry and flood grinding modes. Flood grinding benefited from the cooling, lubrication, and cleaning effects of grinding fluid so that it provided a lower level of wear in terms of blockage and grits pulling off when compared to dry grinding. For the case of progressive increase of the number of active grits and wear flats during grinding applied to diamond grinding tools, Hwang et al. [11] found a reduction in the surface roughness of ground surfaces. Costes et al. [12] demonstrated that the adhesion and the diffusion of species from workpiece materials to CBN tools were the dominant wear mechanisms during machining nickel-based superalloy Inconel 718. This was attributed to the inherent property of nickel-based superalloys that exhibited intense thermal affinity to the tool materials, as described by Klocke et al. [13].

On the other hand, cooling-lubrication modes have a vital effect on controlling the wear mechanisms of grinding tools in grinding operations [14]. In recent years, due to the serious concern regarding environmental problems for machining, the minimum quantity lubrication (MQL) technology has been adopted as a cooling-lubrication mode in machining. Silva et al. [15] showed that MQL technology consumed a minimum quantity of lubricant to offer the needed lubricating effects; this was identified as a low-cost and environment-friendly lubrication technology. Tawakoli et al. [16] showed that MQL technology efficiently reduced the wear of CBN and corundum wheels and improved workpiece surface quality. This can be associated with the oil-mist efficiently penetrating into the grinding contact zone which resulted in the formation of an oil-film that improved the lubrication in machined operations [17]. Salur et al. [18] and Şap et al. [19] reported comparative studies of machinability under dry mode and MQL mode, and experimental results showed that MQL mode provided more effective protection for tools by the hybridized pressured air and greasing effect from the oil-film when compared to dry environment. Additionally, in order to enhance the base oil thermal conductivity and lubricant antifriction aspects, some works [20,21] added nanoparticles, such as graphene, multilayer graphene (MG), Al_2_O_3_ nanoparticles, and diamond nanoparticles, into the base oil, which was termed as the nanofluid [22]. Due to the advantaged properties of MG involving high thermal conductivity, Li et al. [23] adopted MG as lubricating oil additive to investigate the performance of an MG nanofluid during machining. Moreover, Al_2_O_3_ nanoparticles were used as lubricating oil additives to explore the effect of Al_2_O_3_ nanofluid on the grinding performance, as reported by Setti et al. [24], and results showed a reduction in tangential forces and coefficient of friction.

The above literature review reflects that a lot of studies have employed MQL or nanofluid MQL. Despite that MQL and nanofluid MQL offer a potential option to achieve good lubricating effect, the monitoring of process, including tool wear conditions, cannot be ignored. Previous works mainly focused on the evaluation of lubricating property and ground surface quality when grinding nickel-based superalloys adopted various cooling-lubrication modes; however, the wear mechanisms of grinding tools during grinding nickel-based superalloys under different cooling-lubrication modes have yet to be determined. Therefore, the purpose of this study is to investigate the wear mechanisms of CBN grinding tools during grinding nickel-based superalloys under various cooling-lubrication modes such as dry, flood, MQL-PO, MQL-MG, and MQL-Al_2_O_3_ modes. The interaction between the grinding tool and the workpiece is analyzed from a technology perspective. Furthermore, grinding performance in terms of grinding forces, coefficient of friction, and surface profile/roughness/topography after grinding using various types of cooling–lubrication modes are investigated.

## 2. Materials and Methods

### 2.1. Tool, Workpiece, and Machine Tool

**Figure 1:**
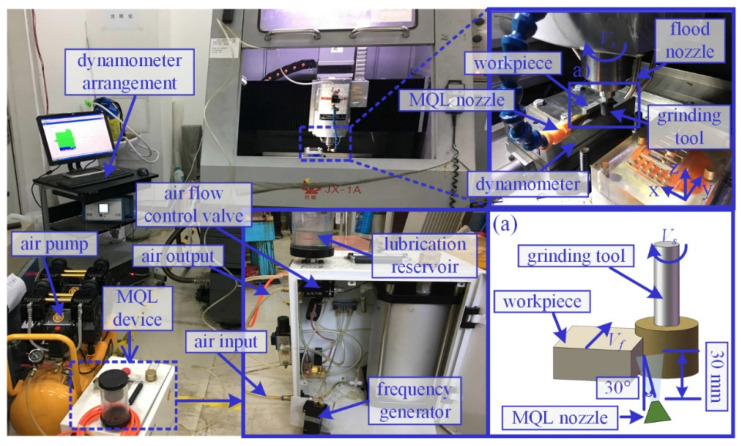
Experimental setup, (**a**) schematic of grinding zone.

All the machined experiments were carried out with the CBN grinding tools (Shanghai Xinlun Superabrasives Co., Ltd, Shanghai, China) on nickel-based single crystal superalloy DD5 (provided by the Institute of Metal Research, Chinese Academy of Sciences, Shenyang, China) on the precision grinder JX-1A (Shenzhen Crab Precision Machinery Equipment Co., Ltd, Shenzhen, China), as shown in Figure 1.

Machining parameters used in the experiments are given in Table 1, which are in accordance with a few preliminary experiments.

**Figure 2:**
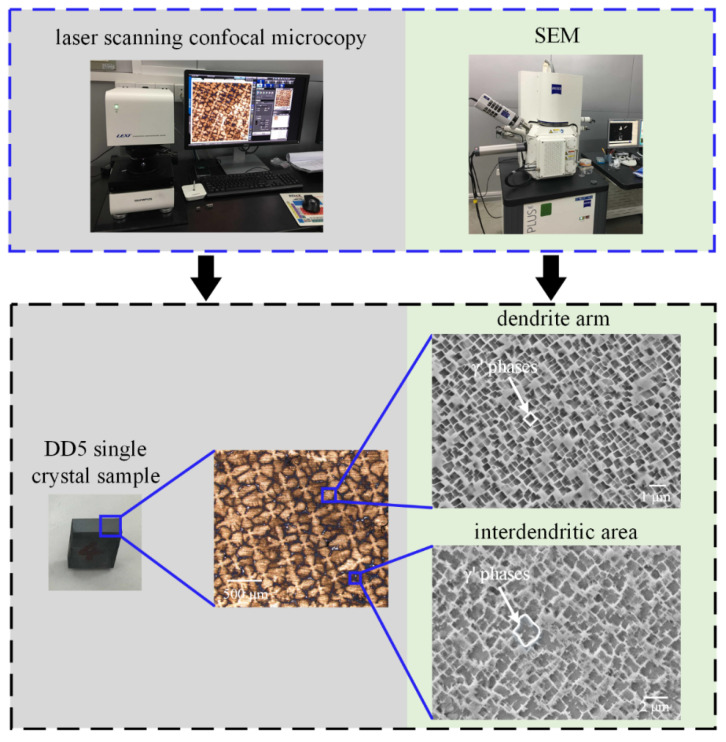
Microstructure of nickel-based single crystal superalloy DD5.

Nickel-based single crystal superalloy DD5 was adopted for grinding experiments, as shown in Figure 2.

The optical microstructure from the confocal microcopy presents the “+” dendrite structure on the (001) plane. Furthermore, it can be observed that no grain boundary appeared on both the optical image and scanning electron microscope (SEM, produced by Carl Zeiss group, Munich, Germany) images, which eliminates massive weakening phases in materials. SEM images present the regular and fine cubic γ’ phases in the dendrite arm and the irregular and coarse γ’ phases in the interdendritic area. The nominal composition of DD5 alloy is given in Table 2 [25]. The mechanical properties of nickel-based single crystal superalloy DD5 are shown in Table 3.

Rectangular block workpieces measuring 10 mm long and 6 mm thick were adopted for proceeding grinding experiments; a large nickel block was cut by an electrical discharge wire machine to obtain rectangular samples. The size of the CBN grinding tool is 16 mm outside diameter and 12 mm height. All grinding experiments were carried out in down cut condition and repeated for five passes. Before proceeding with the experiments, CBN grinding tools were dressed used a diamond dressing roller.

### 2.2. Cooling-Lubrication Modes

Five cooling–lubrication modes, including the dry, flood, MQL-PO, MQL-MG, and MQL-Al_2_O_3_ modes, were adopted in this study. Emulsion in a 5% concentration (95% water) was selected as the grinding fluid for the flood grinding mode. Since palm oil offered good lubrication deriving from its high viscosity and high content of triglycerides [26], palm oil was used as the MQL base oil. The multilayer graphene (provided by Shenzhen Turing Evolution Technology Co., Ltd, Shenzhen, China) and Al_2_O_3_ nanoparticles (provided by Shanghai Chaoqin New Material Technology Co., Ltd, Shanghai, China) mixed with palm oil were selected as the MG nanofluid and Al_2_O_3_ nanofluid, respectively. The technology parameters of multilayer graphene and Al_2_O_3_ nanoparticles are listed in Table 4 and Table 5, respectively.

**Figure 3:**
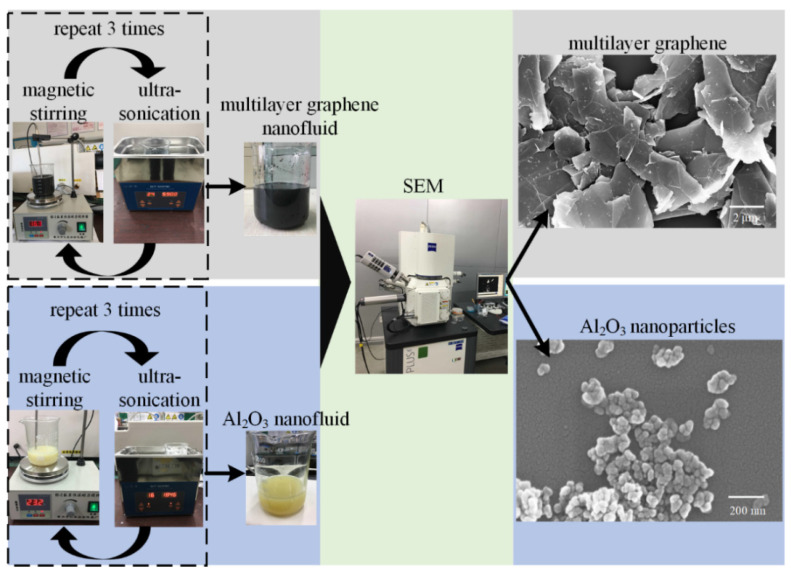
Preparation method for nanofluids.

The nanofluids were prepared by dispersing multilayer graphene and Al_2_O_3_ nanoparticles into palm oil, as shown in Figure 3.

The MG (or Al_2_O_3_ nanoparticles) were mixed into the palm oil, which was stirred using the magnetic stirrer (produced by Changzhou Yineng Experimental Instrument Factory, Changzhou, China) for 30 min first, then we applied ultrasonic waves provided by the ultrasonicator (produced by GuangDong GT Ultrasonic Co., Ltd., Zhongshan, China) through the nanofluids (1 h) to further stabilize the nanofluids. This dispersion process was repeated three times to ensure uniform distribution of the nanofluids in a stable way. Based on the preliminary experiments and the consideration of the tribological performance and stability concerns of nanoparticles, the mass proportions of 0.05 wt% for MG and 0.5 wt% for Al_2_O_3_ nanoparticles were adopted in this study. In order to enhance the stability of nanofluids, the surfactant sodium laurel sulphate (SDS) weighing one-tenth of the weight of a nanoparticle was added to the fluids based on the studies by Singh et al. [27] and Demas et al. [28]. In addition, the microstructures of MG and Al_2_O_3_ nanoparticles can be observed in Figure 3.

The MQL device used in the current experiments is shown in Figure 1. Compressed air was mixed with lubricating oil at the MQL nozzle to spray oil mist and gain entry into the grinding contact zone. The MQL nozzle position is shown in Figure 1a, and the distance of 30 mm and the angle of 30° toward the grinding contact zone were used in this experiment.

### 2.3. Assessment of Tool Wear, Grinding Force, and Surface Quality

Detailed examinations of the CBN grinding tool were conducted with an SEM to evaluate the tool wear under different cooling-lubrication modes. Grinding forces and coefficient of friction are strongly associated with the machine responses between the grinding tool and the workpiece, including tool wear, tribology behavior, and material properties [29,30]. Hence, grinding forces in grinding operations were measured online with a dynamometer (Kistler 9257B, produced by Kistler Group, Winterthur, Switzerland). The instrument was installed with the Dynoware software (Type 2825A, Winterthur, Switzerland) and equipped with an 8-channel charge amplifier (5070A). In order to ensure the reliability of experimental results, each experiment was repeated five times; reported data were the average values of five measurements. After each experiment, the coefficient of friction was calculated using the values of the tangential and normal forces, applying the following equation:

Coefficient of friction (*μ*) = tangential force (*Ft*)/normal force (*Fn*)

On the other hand, the values of workpiece surface roughness (*Ra* and *Rz*) were measured perpendicular to the feed direction with a laser scanning confocal microscope (Olympus OLS4100, produced by Olympus Corporation, Tokyo, Japan); the average values of five different locations on the ground surface were reported as the surface roughness results. In the measurement of surface roughness, 0.8 mm cut-off length (λc) and 0.0025 mm cut-off length (λs) were used. The ground surface topographies were detected with a super-depth microscope (Keyence VHX-1000E, produced by Keyence Corporation, Osaka, Japan).

## 3. Results and Discussion

### 3.1. Wear Mechanism of Tool

**Figure 4:**
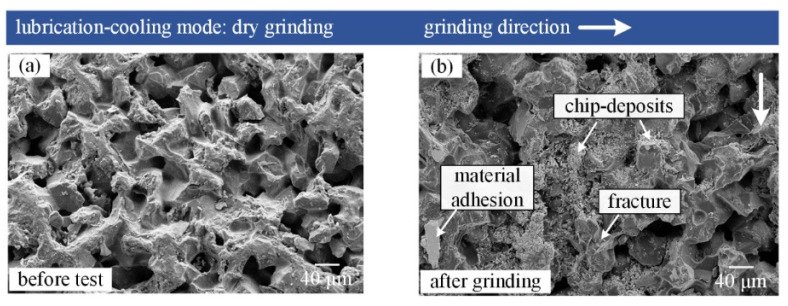
SEM micrographs of grinding tool (**a**) before test, (**b**) after grinding in dry grinding mode.

Figure 4 shows SEM micrographs of the CBN grinding tool in dry grinding mode.

**Figure 5:**
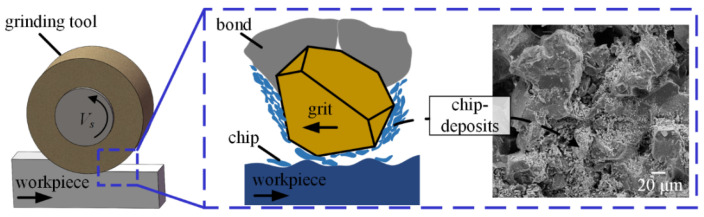
Schematic of the grinding interface in dry grinding mode.

Chip-deposits, material adhesion, and fractures appeared on the surface of the grinding tool. In comparison with the tool surface before the test, a noticeable phenomenon after dry grinding was that chip-deposits not only covered an amount of CBN grits, but also clogged some spaces between grits on the tool surface. The chip-deposits existing on the tool surface resulted in a reduction of the effective protrusion of CBN abrasive grits. Formation mechanism of chip-deposits can be explained with the help of a schematic of the tool-workpiece interface under dry grinding mode, as shown in Figure 5.

In the dry grinding environment without supplying any cooling fluid, alloy chips cannot be effectively transferred from the tool surface, especially for nickel-based superalloys, which have high chemical affinity to grinding tool materials [31]. Meanwhile, dry grinding nickel-based superalloys led to high heat generation in the tool-workpiece contact zone [32], which may further accelerate the chip sticking; eventually, chip-deposits formed on the tool surface with repeated rotation of the grinding tool. A similar result was reported by Wojtewicz et al. [33] while grinding nickel-based superalloys Inconel 718, and they found that the chip-deposits clogged some of the grinding wheel active surface. Therefore, chip-deposits are the principle wear mechanism of grinding tools in grinding of nickel-based superalloys using dry grinding mode. Moreover, such chip-deposits significantly reduce the cutting ability and service life of grinding tools, and cause component damage [34].

**Figure 6:**
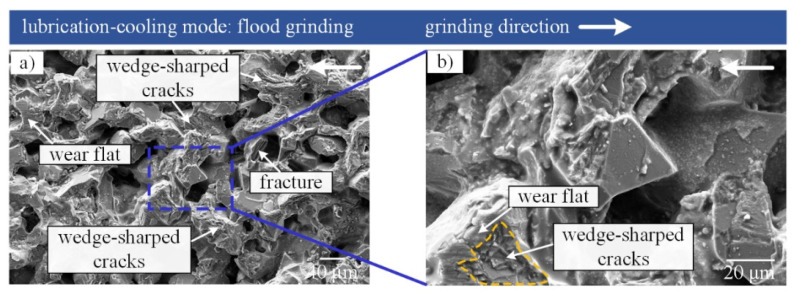
(**a**) SEM micrographs of grinding tool in flood grinding mode, (**b**) enlarged view from the square in (**a**).

In comparison with dry grinding mode, only a small number of chips were observed on the surface of the CBN grinding tool under flood grinding mode, which demonstrates that effective chips transfer occurred on the tool surface resulting from the cooling and flushing behaviors of the grinding fluid (see Figure 6).

**Figure 7:**
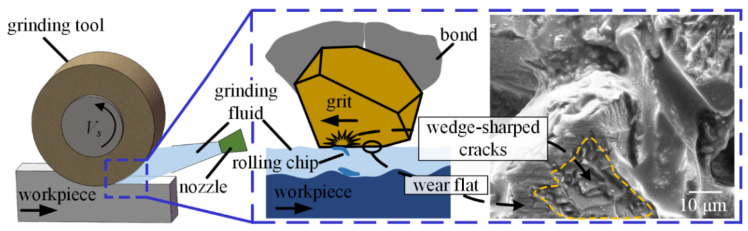
Schematic of the grinding interface in flood grinding mode.

An interesting phenomenon was observed where a number of wedge-shaped cracks appeared on the abrasive grits. The formation mechanism of wedge-shaped cracks can be explained by Figure 7.

It can be observed in Figure 7 that, in the flood cooling environment, lots of grinding fluid impacts the tool-workpiece contact zone and causes chip rolling, which results in rolling chips adhered to the surface of CBN grits. The rolling chips provided repeated pressing behaviors, acting upon CBN abrasive grits, that induced mechanical fatigue and eventually formed wedge-shaped cracks. Furthermore, a number of new cutting edges were provided by the wedge-shaped cracks on CBN grits, which contributed to enhancing the cutting ability of the CBN grinding tool. Wedge-shaped cracks are the principal wear mechanism when grinding nickel-based superalloy under flood grinding mode.

On the other hand, Figure 6 shows that both the wedge-shaped cracks and wear flat can occur on one single CBN grit. Two possible conditions can induce this phenomenon: (1) Wedge-shaped cracks formed on the whole CBN grit at first, then the high-protrusion portions in the wedge-shaped cracks were involved in the interaction of grits-workpiece and formed wear flat in this area. (2) Wear flat formed on the whole abrasive grit at first, then some alloy chips covered the partial wear flat which was induced by the randomness of the interaction between chips, workpiece, and CBN grits; this resulted in wedge-shaped cracks forming on the partial wear flat.

**Figure 8:**
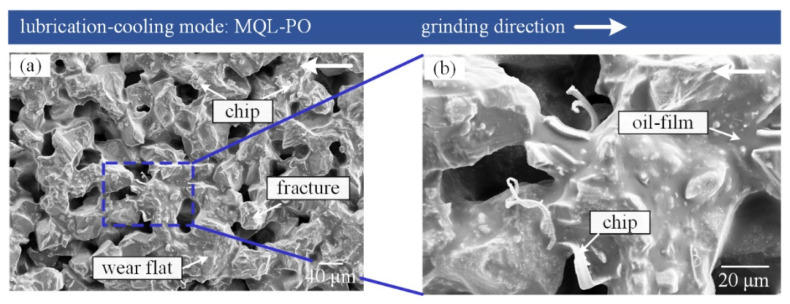
(**a**) SEM micrographs of grinding tool in MQL-PO mode, (**b**) enlarged view from the square in (**a**).

It can be seen from Figure 8 that not only are wear flats, chips, and fractures are visible, but the oil-film also exists on the tool surface under the MQL-PO strategy.

**Figure 9:**
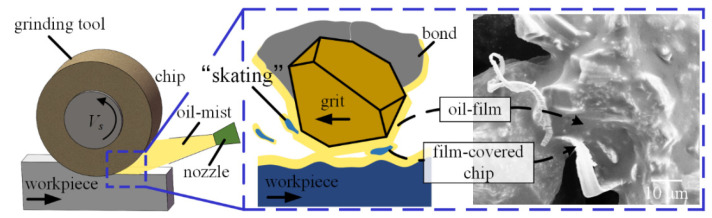
Schematic of the grinding interface in MQL-PO mode.

The oil-film was obviously absent for the dry and flood grinding modes. Palm oil exhibited high lubrication and viscosity, as reported by Wang et al. [26]; high-pressure air drove palm oil mist to penetrate the tool-workpiece contact zone that generates the oil-film on the surface of the grinding tool. The oil-film does not break easily during grinding nickel-based superalloys, as shown in Figure 8b. Figure 9 shows that the oil-film can prevent direct contact between abrasive grits, chips, workpiece, and vitrified bond, thereby retaining the sharpness of CBN grits.

Moreover, film-covered chips adopted an action similar to “skating” to slide over the film-covered tool surface, eventually being removed from the grinding tool with the rotation of the abrasive tool. Accordingly, a small amount of chips exists on the surface of the grinding tool using MQL-PO mode, as shown in Figure 8. Gupta et al. [35] reported that, MQL mode showed an obvious improvement in terms of damaged areas, and chips/material adhered to the cutting edges when compared to dry environment. Attritious wear such as wear flats can be observed by SEM measurement, and its mechanism also can be confirmed by EDS analysis based on composition of elements. Attritious wear is the principal wear mechanism during grinding nickel-based superalloys using the MQL-PO mode due to the formation of lubricated oil-film.

**Figure 10:**
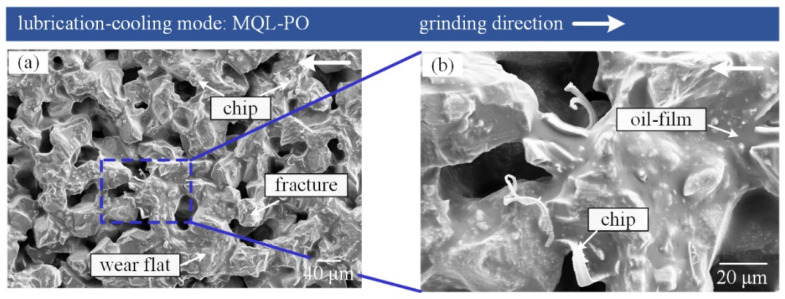
(**a**) SEM micrographs of grinding tool in MQL-MG mode, (**b**) enlarged view from the square in (**a**).

Figure 10 shows SEM micrographs for the CBN grinding tool in MQL-MG mode. The oil-film and chips are visible on the surface of the grinding tool.

**Figure 11:**
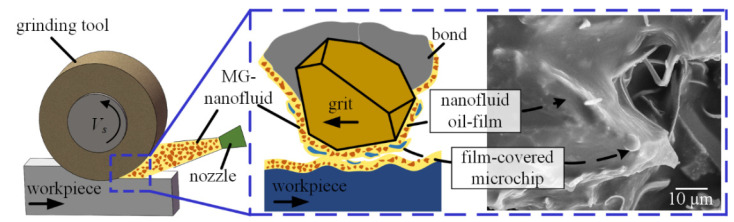
Schematic of the grinding interface in MQL-MG mode.

The purpose for dispersing the graphene into oil is to increase the thermal conductivity of the base oil, which contributes to reducing the temperature in the grinding tool-workpiece contact zone. However, an unexpected and noticeable phenomenon in MQL-MG mode, observed from Figure 10, is that a number of microchips spread over the tool surface, which was rare in the MQL-PO mode. The reason for this phenomenon can be explained with the help of Figure 11.

High-pressure air from the MQL nozzle carried the MG-nanofluid into the tool-workpiece contact zone, which resulted in the formation of the MG-nanofluid oil-film in the interface between the tool and workpiece [27]. Furthermore, Oliveira et al. [36] reported that dispersing graphene into oil can increase the viscosity of nanofluids. Hence, high-viscosity oil-film resulted in the alloy microchips adhering to the tool surface, which cannot be removed from the tool despite the rotation of the grinding tool. Since these microchips participated in the interaction between the CBN grits and workpiece, they may have an adverse effect on surface quality of the workpiece. Therefore, attritious wear with the participation of microchips is the principal wear mechanism during grinding nickel-based superalloys in MQL-MG mode.

**Figure 12:**
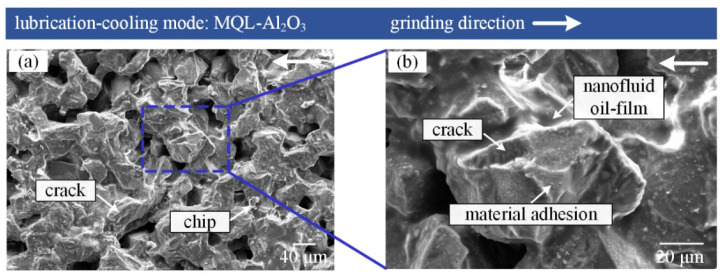
(**a**) SEM micrographs of grinding tool in MQL-Al_2_O_3_ mode, (**b**) enlarged view from the square in (**a**).

Figure 12 presents SEM micrographs of the grinding tool in MQL-Al_2_O_3_ mode.

**Figure 13:**
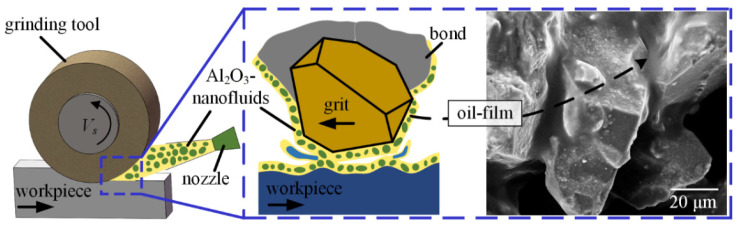
Schematic of the grinding interface in MQL-Al_2_O_3_ mode.

A small amount of chips exists on the surface of the grinding tool using MQL-Al_2_O_3_ mode, and there were not as many microchips as in MQL-MG mode. The Al_2_O_3_ nanofluid oil-film covered the surface of the grinding tool, which resulted in Al_2_O_3_ nanoparticles involved in the interaction between the grinding tool and the workpiece, as described by Kalita et al. [37]. The current experiments adopted Al_2_O_3_ nanoparticles HR = 2700–3000 that can be regarded as a hard phase; they showed a high wear resistance in the frictional process and reduced the actual contact area for the friction pair between the grinding tool and the workpiece [38]. Furthermore, Figure 13 shows that Al_2_O_3_ nanoparticles in the oil-film present a behavior similar to “ball bearings”, which can change sliding friction into sliding-rolling mixed friction at the tool-workpiece interface. Attritious wear is the principal wear mechanism when grinding nickel-based superalloys employing MQL-Al_2_O_3_ mode.

### 3.2. Grinding Force and Coefficient of Friction

**Figure 14:**
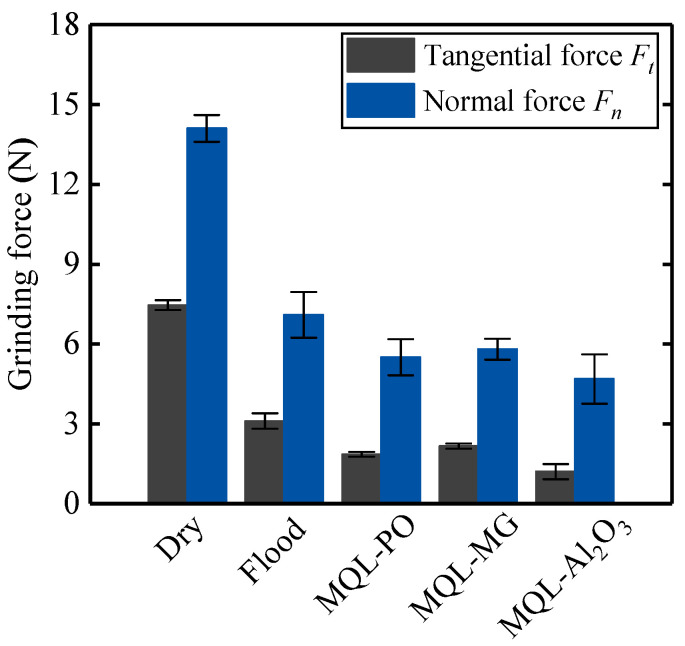
Grinding force against different cooling-lubrication modes.

Figure 14 shows the tangential forces and normal forces (with associated standard deviation) in different cooling-lubrication modes.

Dry grinding mode produced the highest values of tangential force (7.46 N) and normal force (14.1 N), which were found to be 58% and 50%, 75% and 61%, 71% and 59%, and 84% and 67% higher than the flood, MQL-PO, MQL-MG, and MOL-Al_2_O_3_ modes, respectively. High grinding forces in dry grinding mode can be attributed to chip-deposits occurring on the surface of the grinding tool (Figure 4). Chip-deposits covering the abrasive grits not only significantly decreased the number of effective cutting edges, but also generated high temperatures which resulted in the softening of abrasive grits [24]. Salur [39] demonstrated that, when machining nickel-based superalloy Inconel 718 in dry environment, chips or alloy materials adhered to the surface of the tool can act as irregularly shaped cutting edges to participate in the machining process, which can increase the friction and forces. These disadvantages from the dry grinding mode consequently lead to high values of tangential forces and normal forces. A similar result, that dry grinding generated the highest grinding forces for all the different cooling-lubrication modes, was reported by Ibrahim et al. [40].

MQL grinding modes including MQL-PO, MQL-MG, and MQL-Al_2_O_3_ showed relatively low grinding forces, as shown in Figure 14; this can be associated with the formation of the oil-film in the interface between the grinding tool and the workpiece, which offered good lubrication during grinding (Figure 8, Figure 10, and Figure 12). Tawakoli et al. [41] described that effective lubrication from MQL modes can help to preserve the sharpness of cutting edges on abrasive grits, hence decreasing grinding forces. In addition, MQL-Al_2_O_3_ mode produced the lowest values of tangential force (1.2 N) and normal force (4.68 N) in all the tests, this can be attributed to the Al_2_O_3_ nanoparticles having high wear resistance and that they provided the “ball bearing” effect that reduced the friction in the interface between the abrasive grits and the workpiece.

It was observed that flood grinding mode also generated relatively lower grinding forces than dry grinding mode. Unlike the three MQL modes providing the lubricant oil-film, the low grinding forces produced in the flood grinding mode can be attributed to the formation of wedge-fractures on the abrasive grits (Figure 6). The continued generation of wedge-fractures during flood grinding provided an amount of new cutting edges that improved the cutting ability of the grinding tool, which resulted in decreasing grinding forces.

**Figure 15:**
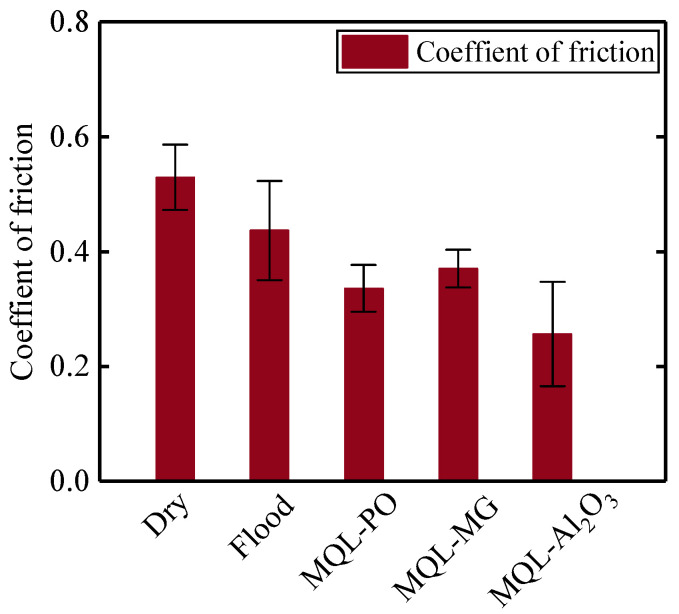
Coefficient of friction against different cooling–lubrication modes.

Interfacial friction in the grinding tool-workpiece contact zone plays an important role for the evaluation of grinding performance. Figure 15 reveals the coefficient of friction in different cooling-lubrication modes.

Dry grinding mode presented the highest coefficient of friction, *μ* = 0.53, which can be associated with chip-deposits on the grinding tool during grinding nickel-based superalloys, as discussed earlier. Formation of chip-deposits decreased the number of effective cutting edges and significantly increased the friction in the interface between the CBN grits and the workpiece. MQL-PO mode provided a 36% reduction in the coefficient of friction when compared to dry grinding mode. This result is mainly due to the formation of a lubricating oil-film that effectively decreased the friction in the grinding contact zone [42]. Yıldırım et al. [43] demonstrated that the oil-film between the tool and the workpiece offered a positive contribution to friction due to the decrease of adhesion that appeared on the tool, which reduced the friction and the rate of tool wear when machining nickel-based superalloys Inconel 625. The lowest coefficient of friction, *μ* = 0.26, was obtained in MQL-Al_2_O_3_ mode, which was 51% lower than dry grinding mode. This demonstrates that addition of Al_2_O_3_ nanoparticles to the base oil provides a significant improvement to the lubricity.

### 3.3. Grinding Surface Quality

**Figure 16:**
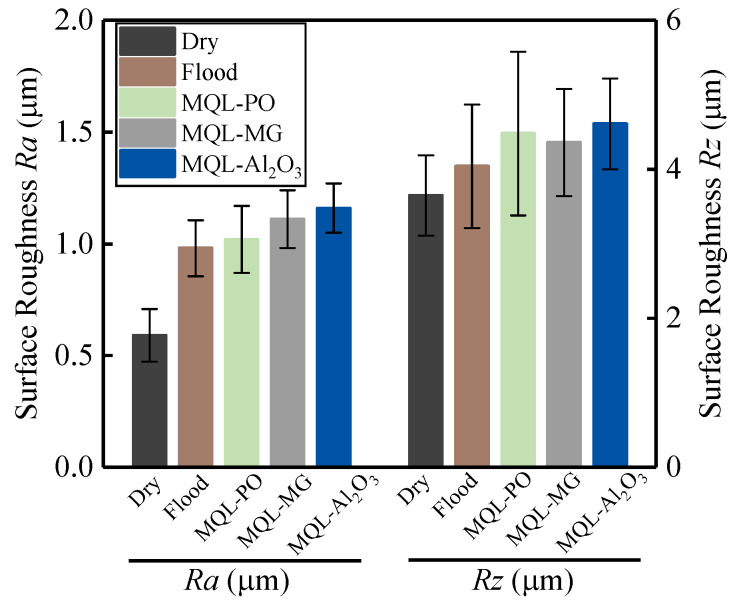
Surface roughness against different cooling-lubrication modes.

Figure 16 shows surface roughness *Ra* and *Rz* when employing different cooling-lubrication modes in grinding of nickel-based superalloys.

The results from Figure 16 suggest that the flood grinding, MQL-PO, MQL-MG, and MQL-Al_2_O_3_ modes generate relatively higher average values of surface roughness when compared to dry grinding mode. Palmer et al. [44] reported that grinding tool topography has a critical impact on ground surface, with the differences between various tool topographies causing the variation in surface responses. The tool topography for flood grinding mode showed new cutting edges generated by the formation of wedge-shaped fractures (Figure 6), which produced uneven ground surface that increased the values of surface roughness. Tool topographies from MQL-PO, MQL-MG, and MQL-Al_2_O_3_ modes, respectively, showed that oil-film, MG-nanofluid oil-film, and Al_2_O_3_-nanofluid oil-film covered the tool surfaces, which maintained the sharpness of abrasive grits. Therefore, relatively higher surface roughness, *Ra* = 1.02 μm, 1.11 μm, and 1.16 μm, were achieved, respectively, by these three MQL modes. Dry grinding mode produced the lowest values of surface roughness of *Ra* = 0.59 μm and *Rz* = 3.65 μm; this can be associated with chip-deposits clogging some areas on the tool surface, which resulted in the uniform protrusion of abrasive grits (Figure 5).

**Figure 17:**
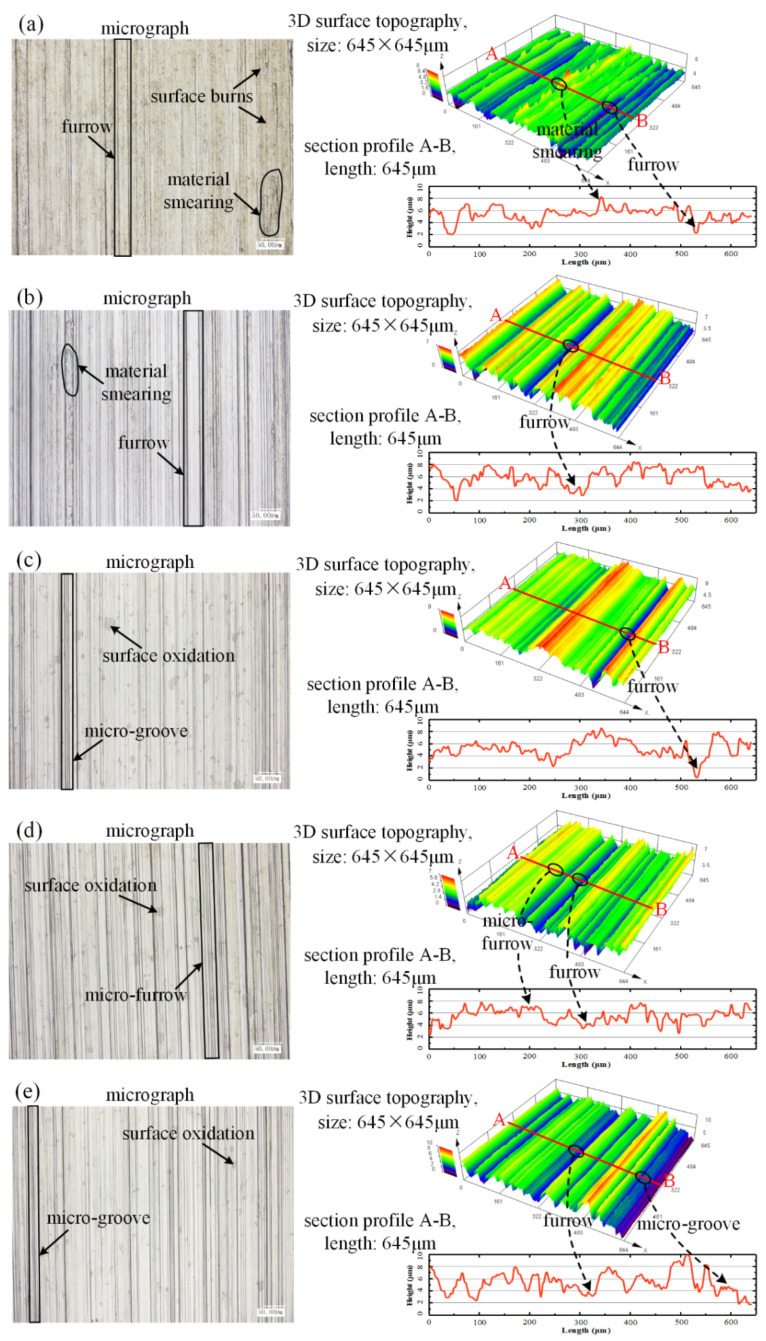
Micrographs, 3D topographies, and cross-section profiles against the five cooling-lubrication modes, including (**a**) dry grinding, (**b**) flood grinding, (**c**) MQL-PO, (**d**) MQL-MG, and (**e**) MQL-Al_2_O_3_.

Surface roughness *Ra* and *Rz* are critical indicators for assessing grinding characteristics and service performance of a workpiece [45]. Figure 17 shows micrographs, 3D topographies, and cross-section profiles in five cooling-lubrication modes. Application of dry grinding mode showed that not only material smearing and furrows, but also surface burns took place on the ground surface. Appearance of surface burns suggests that the abrasive grits covered by chip-deposits raised the temperature in the tool–workpiece contact zone during grinding nickel-based superalloy. Similar results for ground surface burns during grinding nickel-based superalloys have been reported in several works [46,47]. In addition, material smearing bulges on the ground surface and the furrows are sunken, which can also be seen in the 3D topography for dry grinding mode. The cross-section profile in dry grinding mode shows that the curves for material smearing correspond to relatively high peaks; in contrast, they are valleys for furrows. Surface oxidations were visible in MQL-PO, MQL-MG, and MQL-Al_2_O_3_ modes; however, material smearing rarely appeared. Moreover, three MQL modes presented some micro-furrows on the ground surface, thereby indicating that the formation of lubricating oil-film maintains the sharpness of abrasive girts.

## 4. Conclusions

Grinding of difficult-to-cut materials such as nickel-based single crystal superalloy DD5 leads to serious tool damage and low dimensional accuracy over ground surface. Therefore, in the present study, wear mechanisms of CBN grinding tools and grinding performance in grinding of nickel-based superalloys using different cooling-lubrication modes (e.g., dry, flood, MQL-PO, MQL-MG, and MQL-Al_2_O_3_ modes) were investigated. Based on the findings, the following conclusions can be drawn:

1. Chip-deposits were the principal wear mechanism of the CBN grinding tool under dry grinding mode; they covered some areas on the tool surface, which accelerated the tool failure. Dry grinding mode provided the highest tangential force (7.46 N) and normal force (14.1 N), which was found to be 58% and 50%, 75% and 61%, 71% and 59%, and 84% and 67% higher than the flood, MQL-PO, MQL-MG, and MOL-Al_2_O_3_ modes, respectively. MQL-Al_2_O_3_ mode produced the lowest values of tangential force (1.2 N) and normal force (4.68 N) due to the “ball bearing” effect from Al_2_O_3_ nanoparticles that reduced the friction in the interface between the abrasive grits and the workpieces.
2. Wedge-shaped fractures occurred on the CBN grits during grinding nickel-based superalloys when applying flood grinding mode, which provided new cutting edges. Very few chips were observed on the tool surface due to the effective cleaning and cooling behaviors from the grinding fluids.
3. MQL-PO, MQL-MG, and MQL-Al_2_O_3_ modes exhibited the formation of oil-film on the tool surface, which improved the tribological performance in the interface between the tool and the workpiece. The 37%, 30%, and 52% reduction in coefficient of friction were achieved with MQL-PO, MQL-MG, and MQL-Al_2_O_3_ modes when compared to dry grinding mode, respectively.
4. Despite that dry grinding produced the lowest value of surface roughness, 0.59 μm *Ra*, burns were visible on the ground surface. The application of MQL-PO, MQL-MG, and MQL-Al_2_O_3_ modes retained the sharpness of grits, which resulted in a 73%, 88%, and 97% increase in surface roughness, Ra, when compared to dry grinding, respectively.

The knowledge gathered within this study improved the understanding of the wear mechanisms of CBN grinding tools and grinding performance of grinding nickel-based superalloys under various cooling-lubrication modes. In the next step, a novel phenomenological model will be developed based on the experimental findings, which involves the grinding process kinematics and the thermomechanical loading produced at the tool-workpiece contact zone. Meanwhile, the model also may be taken into account to predict the life of CBN grinding tools in grinding nickel-based superalloys.

## Figures and Tables

**Table 1:** Process parameters.

Grinding Type	Plunge Surface Grinding, Down Cut
Type of wheel	CBN320N5V (vitrified bond)
Machine tool	JX-1A grinder
Workpiece material	Nickel-based single crystal superalloy DD5
Workpiece crystallographic orientation	(001) plane [100] orientation
Cooling-lubrication modes	Dry, flood, MQL-PO, MQL-MG, MQL-Al_2_O_3_
Feed rate (*V_f_*)	*V_f_* = 1 mm/s
Depth of cutting (*a_p_*)	*a_p_* = 20 μm
Wheel speed (*V*_s_)	*V*_s_ = 30 m/s
MQL oil	Palm oil
MQL flow rate (*Q*)	*Q* = 120 cm^3^/h
Pressure of air (*P*) in MQL	*P* = 0.4 MPa
Nozzle position (*D*)	*D* = 30 mm
Dresser	Diamond dressing roller
Dressing speed ratio (*q_d_*)	*q_d_* = 0.8
Total depth of dressing(*a_d_*)	*a_d_* = 80 μm

**Table 2:** The nominal composition of nickel-based SX superalloy DD5 (wt%).

W	Ta	Al	Re	Mo	Co	Gr	C	Hf	Ni
5	7	6.2	3	2	7.5	7	0.05	0.15	Bal

**Table 3:** Mechanical properties of nickel-based SX superalloy DD5.

Melting Point	Shrinkage	Hardness	Yield Strength
1368 °C	13.5%	550 HV	1109 Mpa

**Table 4:** Technology parameters of multilayer graphene.

Number of Layers	Mean Diameter (μm)	Layer Thickness (nm)	Specific Surface Area (m^2^/g)	Appearance
6–10	5–50	3.4–8 nm	350–450	Black powder

**Table 5:** Technology parameters of Al_2_O_3_ nanoparticles.

Grain Size (nm)	Specific Surface Area (m^2^/g)	Volume Density (g/cm^3^)	Crystal Form	Appearance
50	58	0.55	α	White powder

## Data Availability

Not applicable.
